# Developing sustainable generalism education in community settings: Insights from the JPCA (Japan Primary Care Association) Conference, 2024

**DOI:** 10.1002/jgf2.770

**Published:** 2025-01-10

**Authors:** Shinji Matsumura, Yoshiko Yamaguchi, Fumiko Okazaki, Fuminao Kitanishi, Satsuki Tomita, Hiroshi Takagi

**Affiliations:** ^1^ Matsumura Clinic Tokyo Japan

## ETHICS STATEMENT

None.

To the Editor,

The principle of being a generalist, or “generalism,” is increasingly recognized as a core component of medical education internationally. Given that primary care medicine is essential in many countries, training medical students and residents in “generalism,” the fundamental aspect of primary care, remains a contemporary challenge.^1^, ^2^ In this context, the revised Model Core Curriculum in Japan for 2022 emphasizes Generalism (GE), and education of “generalism” is gaining even greater importance.^3^

Although most medical education occurs at higher‐level institutions and hospitals, educational resources in community settings are comparatively scarce. Various barriers to community‐based education have been identified, including the limited number of faculty, the dual burden of clinical and teaching responsibilities, and the lack of effective educational materials for learners and supervisors. These challenges hinder the consistent quality of “generalism” education.^4^ Some countries have introduced “community preceptors,” experienced clinicians serving as teaching faculty. Although advanced models exist, further expansion and multifaceted support are needed.^5^

To promote postgraduate and undergraduate “generalism,” education in the community, the authors conducted an interactive discussion and brainstorming session at the 15th Academic Conference of the JPCA (Japan Primary Care Association) in May 2024. During this session, we gathered various perspectives on future community education programs in the context of ‘generalism’ from primary care providers, university faculty, local supervisors, residents, and medical students, all of whom play a crucial role in this endeavor.

The suggestions for future education in the community from the session include:

1. A generalism curriculum that is feasible and sustainable for both learners and supervisors: one‐week programs that are easily adaptable, AI‐driven tools to support personalized learning and reduce supervisor workload, and flexible education packages for diverse needs and schedules.
2. Formation of teaching teams in the community through effective faculty development: establishing multidisciplinary teams of healthcare professionals, providing practical teaching guides for community settings, and encouraging cross‐institutional collaboration to share best practices.
3. Development of shared educational tools: E‐learning materials for common preparatory learning, Virtual Reality (VR) programs to simulate community‐based activities, and AI‐powered tools for sharing and applying educational resources across institutions.

As community preceptors, the authors believe that urgent curriculum development and validation in the local context of community medicine and ‘generalism’ are essential. Such efforts will not only address current challenges but also ensure the sustainability and effectiveness of medical education in community settings.

## AUTHOR CONTRIBUTIONS

SM conceptualized and drafted the manuscript with input from all authors. All authors reviewed and approved the final version of the manuscript for publication.

## CONFLICT OF INTEREST STATEMENT

The authors have stated explicitly that there are no conflicts of interest in connection with this article.
